# Case Report: Refractory systemic nmastocytosis with AML1::ETO+ acute myeloid leukemia driven by rare KIT mutation: remarkable therapeutic efficacy of avapritinib

**DOI:** 10.3389/fped.2025.1646001

**Published:** 2025-10-10

**Authors:** Song Xue, Man Chen, Hui-peng Sun, Xing-yu Cao

**Affiliations:** ^1^ Department of Bone Marrow Transplant, Beijing Lu Daopei Hospital, Beijing, China; ^2^ Division of Pathology & Laboratory Medicine, Beijing Lu Daopei Hospital, Beijing, China; ^3^ Department of Bone Marrow Transplant, Hebei Yanda Lu Daopei Hospital, Langfang, China

**Keywords:** systemic mastocytosis (SM), AML1::ETO, KIT activating mutations, allogeneic hematopoietic stem cell transplantation, avapritinib

## Abstract

This article describes two pediatric SM with AML1::ETO+ AML patients induced by novel KIT exon11 mutations not previously documented in medical literature. Both patients underwent allogeneic hematopoietic stem cell transplantation, but subsequently developed refractory disease progression unresponsive to multiple salvage regiments. Strikingly, avapritinib intervention achieved unprecedented clinical responses in these complex cases.

## Introduction

Systemic mastocytosis (SM) represents a clonal neoplasm characterized by abnormal proliferation of mast cells (MCs) in extracutaneous tissues.The disease comprises a clinicopathologic spectrum manifesting distinct clinical phenotypes and heterogeneous prognostic profiles (1). In the Mayo Clinic cohort, SM with an associated hematologic neoplasm (SM-AHN) consistuted the second most frequent disease variant, accounting for 40% of the total cases (2), with chronic myelomonocytic leukemia (CMML) being the predominant syndrome within the myelodysplastic/myeloproliferative neoplasms (MDS/MPN) category. Other concomitant malignancies included myelodysplastic syndromes (MDS), acute myeloid leukemia (AML), and rarely lymphoproliferative disorders (3). KIT mutations constitute driver events in SM pathogenesis and are mechanistically implicated in AML1-ETO+ AML, explaining their frequent co-occurrence.While the KIT D816V mutation predominates in adult SM (>80% prevalence), pediatric SM exhibits markedly lower D816V incidence coupled with higher frequencies of non-canonical KIT variants (1). Patients harboring SM with AML1::ETO+ AML face dismal prognoses and formidable therapeutic challenges (4–6). As a novel type I tyrosine kinase inhibitor, avapritinib potently targets both wild-type and mutant forms of KIT (exon11/17) and PDGFRA.Here, we report two pediatric SM with AML1::ETO+ AML patients induced by novel KIT exon11 mutations not previously documented in medical literature. Both patients underwent allogeneic hematopoietic stem cell transplantation (allo-HSCT), but subsequently developed refractory disease progression unresponsive to multiple salvage regiments. Strikingly, avapritinib intervention achieved unprecedented clinical responses in these complex cases.

## Case presentation

### Case 1

In July 2023, a 9-year-old male patient presented with cutaneous ecchymoses, prompting bone marrow (BM) evaluation at a regional hospital. Morphologic analysis revealed hypercellular marrow with myeloblast predominance (90%). Flow cytometry (FCM) immunophenotyping confirmed 61% leukemic blasts expressing CD45/CD13/CD34/CD117/CD33/HLA-DR/CD38. Cytogenetic and molecular studies identified: (1) AML1::ETO fusion transcript; (2) pathogenic KIT exon 11 mutation (c.1721_1762dup, p.T574_N587dup; VAF 13%) and SRSF2 exon1mutation(c.284C>A:p.P95H,VAF 10.3%) by NGS; and (3) a complex karyotype-46, XY, t(1;9)(p36;q32), t(8;21) (q22;q22) [19]/46,XY[1].First-line induction with idarubicin/cytarabine (IA) achieved hematologic complete remission (CR). The patient was subsequently transferred to a tertiary referral center for consolidation therapy. Post-induction minimal residual disease (MRD) assessment demonstrated: 0.21% aberrant CD34+/CD117+/CD33+/CD38+/HLA-DR+/CD19- myeloid precursors by FCM and quantitative AML1::ETO transcript elevation (123.4%).Between September 2023 and March 2024, multidrug consolidation regimens were administered sequentially: HAA (homoharringtonine 3 mg d1–7/aclarubicin 10 mg d1–3/cytarabine 150 mg d1–7), IA, CLAG (cladribine/cytarabine/G-CSF), venetoclax/azacitidine + HA (homoharringtonine 2 mg d1–7/cytarabine 100 mg d1–7), ivosidenib/interferon/chidamide/decitabine, and venetoclax/decitabine + HA(homoharringtonine 2 mg d1–7/cytarabine 100 mg d1–7). Serial AML1::ETO quantification revealed fluctuating transcript levels (1.8%–16.6%). Haploidentical allo-HSCT was performed in May 2024 (pre-transplant AML1::ETO: 1.2%). Post-HSCT monitoring detected rising AML1::ETO transcript levels (+30d: 0.3%; + 60d: 0.8%). Azacitidine/interferon maintenance failed to suppress molecular relapse (AML1::ETO: 1.3% at 5 months post-HSCT). Given disease progression, the patient initiated subsequent therapeutic intervention at our institution.

**Figure 1:**
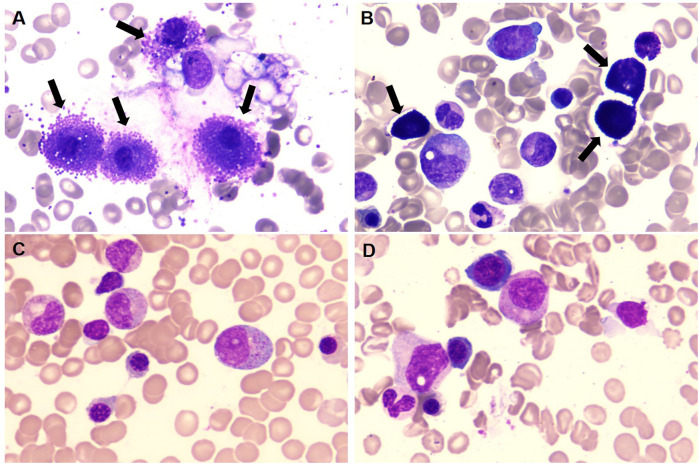
**(A,B)** prior to initiating avapritinib treatment, BM morphology revealed a significant increase in MCs (black arrow), which were clustered in their distribution. **(C,D)** Following avapritinib treatment, abnormal MCs became undetectable on BM aspirate smears. (Wright's stain ×100).

**Figure 2:**
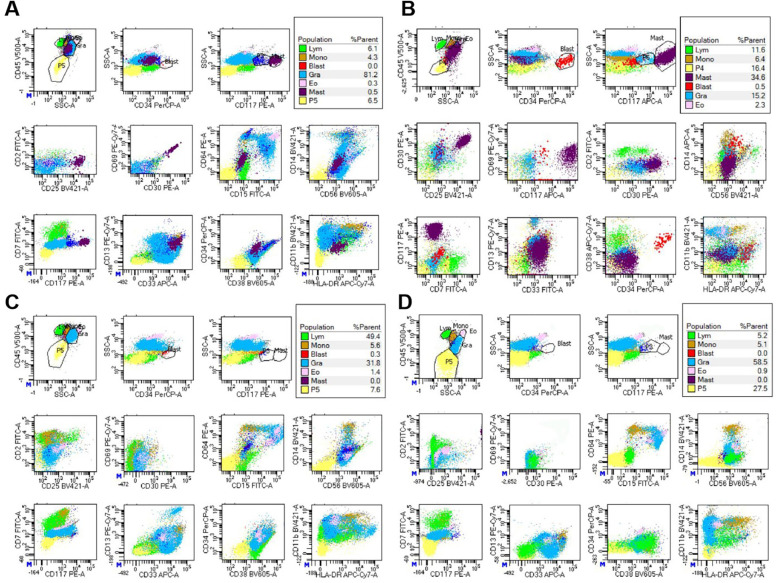
FCM analysis of BM revealed the presence of abnormal MCs before avapritinib treatment, designated as the “purple group”. **(A)** for case 1, abnormal MCs accounted for 0.5% of nucleated cells. These cells demonstrated increased SSC, enhanced CD117 expression, abnormal CD25 and CD30 expression, **(B)** for case 2, abnormal MCs accounted for 34.6% of nucleated cells. These cells demonstrated increased SSC, enhanced CD117 expression, abnormal CD25 and CD30 expression. **(C,D)** Following avapritinib treatment, FCM of the bone marrow confirmed the absence of abnormal MCs.

Institutional re-evaluation confirmed morphologic remission and FCM-negative MRD, though inter-laboratory variance was noted (AML1::ETO: 0.126%).A salvage regimen comprising CAG (cytarabine 20 mg d3–16/aclarubicin 10 mg d3–10/G-CSF 150 ug d3–10) administered concomitantly with decitabine (20 mg d1–5) and olaparib(150 mg bid d1–16) was instituted following established protocols (7), this was subsequently followed by donor stem cell infusion (DSI). BM evaluation at +30d post-treatment demonstrated hypoplasia, clustered abnormal MCs proliferation (40% of total MCs, Figure 1A), aberrant MCs by FCM (0.5%,enhanced CD117 expression, abnormal CD25 and CD30 expression, Figure 2A), and AML1::ETO escalation to 1.561%, meeting diagnostic criteria for SM-AHN (1). Following salvage failure, avapritinib (100 mg daily) was initiated on December 18, 2024. Therapy was well-tolerated. Day +14 post avapritinib assessments confirmed morphologic remission (Figure 1C), FCM-negative MRD (Figure 2C), and undetectable AML1::ETO. The favorable safety profile of avapritinib allowed for sustained, intermittent maintenance therapy, with dose adjustments based on serial hematologic assessments (WBC and platelet counts) until the end of the follow-up period (July 31, 2025). Sustained molecular remission continues under avapritinib maintenance.

### Case 2

In July 2023, a 4-year-old male presented with recurrent fever and arthralgia. Initial bone marrow evaluation at a regional institution revealed myeloblast hyperplasia (21.0% cellularity). FCM immunophenotyping identified 19.5% aberrant myeloid precursors expressing CD45/CD117/HLA-DR/CD33/CD38/CD13/cMPO/cBCL-2 with partial CD11b co-expression. Molecular studies demonstrated elevated AML1::ETO transcript levels (127.4%) and a pathogenic KIT exon 11 insertion (c.1713_1714insGACCCACCTACCCCACCC, p.I571_D572insDPPTPP; VAF 33.7%). Cytogenetic analysis revealed an abnormal clone with 45,X,-Y,t(8;21)(q22;q22)[20]/46,XY[2], confirming AML1::ETO-positive AML.

First-line induction with MA regimen (mitoxantrone/cytarabine) failed to achieve morphologic response, prompting salvage therapy with azacitidine/cytarabine/dasatinib/venetoclax.Persistent disease was evident by FCM (1.27% residual blasts) and escalating AML1::ETO transcript levels (186.6%). Subsequent intensive chemotherapy with etoposide/high-dose cytarabine/dasatinib/venetoclax induced morphologic remission, with FCM-negative and AML1::ETO reduction to 12.9%. Sequential consolidation therapies (HA/MA/EA/decitabine combined with dasatinib/venetoclax) yielded fluctuating transcript levels (3.13-10.3%). In June 2024, haploidentical allo-HSCT was performed (AML1::ETO pre-HSCT: 3.02%). Post-transplant monitoring detected persistent molecular disease (+30d: 2.95%; + 60d: 6.89%). Six cycles DSI and venetoclax failed to suppress leukemia resurgence (AML1::ETO: 10.43% at +5 month). Given disease progression, the patient initiated subsequent therapeutic intervention at our institution.

Reassessment at our center in November 2024 confirmed morphologic CR, though FCM detected 0.36% aberrant MCs with AML1::ETO at 2.043%. Salvage therapy with cladribine/ decitabine/ chidamide induced prolonged myelosuppression. Day +30 post-treatment evaluation revealed abnormal MCs hyperplasia (31% of total MCs, clustered distribution, Figure 1B), FCM-confirmed aberrant MCs (34.6%,enhanced CD117 expression, abnormal CD25 and CD30 expression, Figure 2B), and AML1::ETO elevation to 20.383%, meeting diagnostic criteria for SM-AHN. Following refractory disease progression, avapritinib (100 mg daily) was initiated on January 2, 2025. By Day +14 post avapritinib, morphologic remission was confirmed (Figure 1D) with FCM-negative MCs (Figure 2D) and remarkable reduction of AML1::ETO (0.004%). The favorable safety profile permitted sustained intermittent avapritinib maintenance therapy, with dose modulation based on serial hematologic monitoring (WBC and platelet counts), through the end of follow-up (July 31, 2025).Written informed consent for publication of this report and accompanying images was obtained from the parents of the two patients.

## Discussion

SM with AML1::ETO + AML often exhibits a suboptimal response to standard induction chemotherapy, demonstrating frequent primary resistance to the treatment (5). AML with the AML1::ETO fusion gene is typically associated with favorable clinical outcomes, characterized by high remission rates and prolonged survival. Roughly 25% of AML cases with the AML1::ETO fusion also harbor KIT mutations, which are associated with a less favorable prognosis (8). A previous retrospective study has shown that 10% of AML patients with the AML1::ETO fusion gene exhibit concurrent SM (9). A multicenter retrospective study, conducted in China between January 2009 and December 2022, identified 24 cases of SM with AML1::ETO + AML across 16 centers, as well as 212 cases of AML1::ETO-positive AML harboring KIT mutations during the same period (6). Despite the presence of the KIT D816V mutation not serving as an indicator of SM in the AML1::ETO + AML subgroup (10), according to the two retrospective studies cited, the incidence of AML1::ETO + AML cases with KIT mutations concurrent with mastocytosis has been underestimated. Based on existing data, we hypothesize that the coexistence of SM may exacerbate adverse effects of KIT on prognosis. Therefore, we propose to repeat bone marrow aspiration and biopsy during the cytoreductive phase subsequent to chemotherapy, as it enhances the detection rate of SM, particularly suited for patients exhibiting poor response to therapy.

Allo-HSCT significantly improves the prognosis of patients with SM with AML1::ETO + AML, supporting its strong consideration for this patient cohort (5, 6, 11). Allo-HSCT demonstrates promising outcomes in the treatment of SM-AHN, with a 3-year overall survival (OS) rate of 74% (12).

However, the post-transplantation efficacy for SM with AML1::ETO + AML patients remains suboptimal, as relapses post-transplantation often result in treatment failure.The persistence of MCs in patients undergoing allo-HSCT represents an intriguing clinical observation (5). A recent retrospective study revealed that the progression-free survival (PFS) after allo-HSCT for SM-AML is only 0.7 years (13).The absence of a KIT D816V mutation adversely affected PFS post allo-HSCT. The findings of our study are comparable to those reported in this research. Both patients underwent allo-HSCT. Nevertheless, both patients experienced disease progression shortly after transplantation.Furthermore, their response to various salvage therapies was poor. Hence, initiating appropriate post-transplant maintenance therapy, such as tyrosine kinase inhibitors (TKIs), is a reasonable choice for treatment.

Recently, small-molecule TKIs targeting KIT have demonstrated promising clinical efficacy (1, 14, 15).

Avapritinib has obtained FDA approval as a first-line therapy for adult patients with advanced SM, following positive results from two clinical trials: EXPLORER (NCT02561988) (16) and PATHFINDER (NCT03580655) (17). But clinical trials of avapritinib for SM have mainly been conducted in patients harboring the KIT D816 mutation. Therefore, therapeutic efficacy reports have predominantly centered on this patient cohort. While avapritinib is theoretically efficacious against KIT mutations in exons 11 and 17, data on its application in patients lacking the KIT D816 mutation are currently scarce. *in vitro* studies have demonstrated that the common N822K mutation in exon 17 of KIT exhibits a marked decrease in sensitivity to avapritinib compared to the D816V mutation. Recent literature indicates that avapritinib demonstrates suboptimal efficacy against KIT exon 11 mutations (18). We report the cases of two patients with rare KIT exon 11 mutations who, despite relapsing post-transplantation, demonstrated significant therapeutic responses to avapritinib. These findings offer valuable insights into the use of avapritinib in this patient group. The profound refractoriness observed in this cohort—characterized by progression despite allo-HSCT—mandates therapeutic innovation. We propose that frontline incorporation of avapritinib into conventional chemotherapy backbone (induction/consolidation phases) might overcome early resistance mechanisms. Subsequent allo-HSCT during molecular remission could then consolidate these gains, potentially transforming the disease trajectory.

## Data Availability

The original contributions presented in the study are included in the article/Supplementary Material, further inquiries can be directed to the corresponding author.
