# Supplementary material for: Case Report: Refractory systemic nmastocytosis with AML1::ETO+ acute myeloid leukemia driven by rare KIT mutation: remarkable therapeutic efficacy of avapritinib
Source: Front Pediatr. 2025 Oct 10;13:1646001. doi: 10.3389/fped.2025.1646001 (PMC12549296; doi:10.3389/fped.2025.1646001)
Supplement: Supplementary file 1 [file Table1.docx]

Table1 World Health Organization 5th Edition Refined major and minor systemic mastocytosis criteria and the patient characteristics

| ****Specific diagnostic criteria**** | Case 1  initial diagnosis | Case 1  modify diagnosis | Case 1  initial diagnosis | Case 1  modify diagnosis |
| --- | --- | --- | --- | --- |
| Major criterion：Multifocal dense infiltrates of mast cells (≥15 mast cells in aggregates) in bone marrow biopsies and/or in sections of other extracutaneous organ(s) | NA | NA | NA | NA |
| Minor criteria 1：≥25% of all mast cells are atypical cells (type I or type II) on bone marrow smears or are spindle-shaped in mast cell infiltrates detected in sections of bone marrow or other extracutanous organs | NA | Yes | Yes | Yes |
| Minor criteria 2：KIT-activating KIT point mutation(s) at codon 816 or in other critical regions of KIT in bone marrow or another extracutaneous organ | Yes | Yes | Yes | Yes |
| Minor criteria 3：Mast cells in bone marrow, blood, or another extracutaneous organ express one or more of: CD2 and/or CD25 and/or CD30 | NA | Yes | Yes | Yes |
| Minor criteria 4：Baseline serum tryptase concentration > 20 ng/mL (in the case of an unrelated myeloid neoplasm, an elevated tryptase does not count as an SM criterion. In the case of a known HαT, the tryptase level should be adjusted | NA | NA | NA | NA |

*Abbreviations:* NA: None assessed.

If at least 1 major and 1 minor or 3 minor criteria are fulfilled the diagnosis is SM
